# Relevance of Bacteriophage 933W in the Development of Hemolytic Uremic Syndrome (HUS)

**DOI:** 10.3389/fmicb.2018.03104

**Published:** 2018-12-13

**Authors:** Manuel E. Del Cogliano, Alipio Pinto, Jorge Goldstein, Elsa Zotta, Federico Ochoa, Romina Jimena Fernández-Brando, Maite Muniesa, Pablo D. Ghiringhelli, Marina S. Palermo, Leticia V. Bentancor

**Affiliations:** ^1^Laboratorio de Ingeniería Genética y Biología Celular y Molecular, Universidad Nacional de Quilmes, Buenos Aires, Argentina; ^2^Laboratorio de Neurofisiopatología, Departamento de Fisiología, Facultad de Medicina, Instituto de Fisiología y Biofísica Bernardo Houssay (CONICET), Universidad de Buenos Aires, Buenos Aires, Argentina; ^3^Laboratorio de Fisiopatogenia, Departamento de Fisiología, Facultad de Medicina, Instituto de Fisiología y Biofísica Bernardo Houssay (CONICET), Universidad de Buenos Aires, Buenos Aires, Argentina; ^4^Laboratorio de Patogénesis e Inmunología de Procesos Infecciones, Instituto de Medicina Experimental (CONICET), Academia Nacional de Medicina, Buenos Aires, Argentina; ^5^Microbiology Section, Department of Genetics, Microbiology and Statistics, Faculty of Biology, University of Barcelona, Barcelona, Spain

**Keywords:** bacterio(phages), hemolytic uremic syndrome, Shiga toxin (Stx), Shiga toxigenic *E. coli* (STEC), animal model

## Abstract

Hemolytic uremic syndrome (HUS), principally caused by shiga toxins (Stxs), is associated with Shiga toxin-producing *Escherichia coli* (STEC) infections. We previously reported Stx2 expression by host cells *in vitro* and *in vivo*. As the genes encoding the two Stx subunits are located in bacteriophage genomes, the aim of the current study was to evaluate the role of bacteriophage induction in HUS development in absence of an *E. coli* O157:H7 genomic background. Mice were inoculated with a non-pathogenic *E. coli* strain carrying the lysogenic bacteriophage 933W (C600Φ933W), and bacteriophage excision was induced by an antibiotic. The mice died 72 h after inoculation, having developed pathogenic damage typical of STEC infection. As well as renal and intestinal damage, markers of central nervous system (CNS) injury were observed, including aberrant immunolocalization of neuronal nuclei (NeuN) and increased expression of glial fibrillary acidic protein (GFAP). These results show that bacteriophage 933W without an *E. coli* O157:H7 background is capable of inducing the pathogenic damage associated with STEC infection. In addition, a novel mouse model was developed to evaluate therapeutic approaches focused on the bacteriophage as a new target.

## Introduction

During infection with Shiga toxin-producing *E. coli* (STEC), which harbors the temperate bacteriophage 933W (Plunkett et al., 1999), phages undergo excision and replication and Shiga toxin (Stx) is expressed and released. The free bacteriophages are then able to infect other susceptible bacteria in the gut, exacerbating bacteriophage replication and Stx production (Muniesa and Schmidt, 2014). Bacteriophage excision is linked to the SOS response of bacteria (Kimmitt et al., 2000). Several antibiotics, such as mitomycin C and quinolones, including ciprofloxacin, are contraindicated in STEC infections, because at antimicrobial levels above those required to inhibit bacterial replication they cause DNA damage. The subsequent SOS response has the undesired side effect of simultaneously triggering phage production and expression of *stx*_2_ genes. Additionally, it has been reported that an STEC strain mutated in the cleavage mechanism was unable to induce renal disease and lethality in a mouse model (Tyler et al., 2013).

STEC strains present several pathogenic properties that increase injury in the gut, including the typical A/E lesion, which is produced by several proteins encoded in a pathogenicity island called LEE (Locus of Enterocyte Effacement) (Kaper, 1998; Sperandio et al., 1998). In a previous study on the role of Stx-encoding bacteriophages in STEC infection, we showed that the use of chitosan as an anti-bacteriophage agent led to a decrease in the mortality rate of infected mice (Amorim et al., 2014). In the current work, mice infected with non-pathogenic *E. coli* C600, lysogenic for the Stx bacteriophage 933W, developed renal, intestinal and brain damage typical of STEC infection, indicating that the pathogenic characteristics of the STEC strain, although important, are not essential for the development of hemolytic uremic syndrome (HUS). These results highlight the importance of bacteriophage 933W in HUS development, supporting the hypothesis that anti-bacteriophage agents can provide a new therapeutic approach against STEC infections.

## Materials and Methods

### Strains and Inoculums Preparation

*E. coli* C600Φ933W and *E. coli* C600 were grown in LB media for 16 h at 37°C in agitation. The overnight cultures were centrifuged at 3,000 × g for 15 min. The pellet was washed and resuspended in sucrose 20% at a dose of 2 × 10^8^ CFU/100 μl/mouse.

Bacteriophage induction was done by treatment with a mix of ciprofloxacin (100 μg/mouse) and mitomycin C (5 μg/mouse), which was intragastrically inoculated 30 min post-infection.

### Bacterial Growth Curve

Strains were grown overnight (ON) in LB medium at 37°C with agitation. The ON cultures were diluted 1:100 in LB in 250 ml Erlenmeyer flasks with a final volume of 50 ml. Samples were taken every hour for 6 h and ON (T0,T1,T2,T3,T4,T5,T6,TON) and the optical density at 600 nm of each sample was measured.

### Anti-Bacteriophage Polyclonal Antibodies

Balb/c mice were immunized with inactivated bacteriophages. Bacteriophages were prepared by formalin treatment. Briefly, bacteriophages were incubated overnight in 2% formalin and then dialyzed extensively against PBS. Bacteriophages were purified as previously reported (Del Cogliano et al., 2017). Mice were immunized with bacteriophages emulsified in Freund's complete (initial immunization) or incomplete (subsequent immunizations) adjuvant. Mice received inactivated bacteriophages three times at biweekly intervals. Sera were obtained 15 and 30 days post immunization, tittered and kept at −20°C.

### Mice Infection

C57BL/c mice were bred in the animal facility of the Institute of Experimental Medicine, (IMEX), Academia Nacional de Medicina, Buenos Aires, Argentina. Mice (17–20 days of age, 8–11 g of body weight) were maintained under a 12-h light–dark cycle at 22 ± 2 C and fed with standard diet and water *ad libitum*. Mice were used immediately after weaning.

The experiments performed herein were approved by the IMEX Animal Care Committee in accordance with the principles set forth in the Guide for the Care and Use of Laboratory Animals (National Institute of Health, 1985). After 4 h of starvation, weaned mice were divided into two groups. One group was inoculated intragastrically via a stainless steel canula (model 7.7.1, 0.38 mm 9 22G) (Harvard Apparatus, Holliston, MA) with a single dose of 100 μl of *E. coli* C600:933W strain (2 × 10^8^ CFU/100 μl/mice). The control group was inoculated with *E. coli* C600 strain following the same approach.

Two doses of antibiotics were administered at 5 and 30 min after infection with a mix of 100 μg of ciprofloxacin (Roemmers) and 5 μg of mitomycin C (Sigma Aldrich). Food and water were provided to mice *ad libitum* 4 h after inoculation.

### Histological Studies

For histological analysis, mice were sacrificed 72 h after infection and subjected to necropsy. Mice were transcardially perfused with PBS in order to completely remove the blood, followed by 5% buffer-formaldehyde. Kidneys, small and large intestines and brains were removed, sectioned, fixed in 5% buffer-formaldehyde and paraffin-embedded. Organ sections of paraffin-embedded tissues were stained with hematoxylin and eosin (H&E) and examined by light microscopy. Tubular injury was evaluated by the presence of alterations in the tubular epithelium, basement membrane integrity and necrosis. Vascular interstitial congestion was also assessed. Another group of mice were sacrificed for immunofluorescence analysis. Mice were anesthetized with pentobarbital (100 mg/kg) and perfused transcardially with 0.9% NaCl solution followed by 4% paraformaldehyde in 0.1 M phosphate buffer solution [fixative per animal weight (ml/g)]. Brains were removed from the skull, and post-fixed in the same fixative solution for 2 h. Brain sections were cut on a cryostat. Serial 20-μm-thick coronal sections were obtained and collected in 0.1M phosphate buffer solution. The brain floating sections obtained were processed for bacteriophage, Stx, GFAP or NeuN immunofluorescence.

### Immunofluorescence of Stx2, Bacteriophage, NeuN, CC1, and GFAP

The brains were processed in a cryostat, and the sections (20 μm each) were kept at 20° C in a cryopreservant solution (50% of PBS plus 30% ethylene glycol plus 20% glycerol) until the day the different immunofluorescence assays were carried out. After several rinses with 10 mM PBS, brain floating sections were permeabilized with 0.1% Triton X-100 and blocked with normal goat serum 10% (Sigma, St. Louis, MO, USA) with the same buffer solution for 1 h, and immediately incubated (at 4°C for 48 h) with different primary antibodies (1:500): rabbit anti-Stx2 antibody (1/50; 1.35 mg/ml), mouse anti-bacteriophage 933W, mouse anti-NeuN (Millipore, Temecula, CA, USA), rabbit anti-CC1 (Abcam, Cambridge, UK) or rabbit anti-glial fibrillary acidic protein (GFAP, Dako, Glostrup, Denmark).

After several rinses with 10mM PBS-X-100 0.025%, the sections were incubated with the secondary antibodies (1:500) goat IgG anti-Rabbit Alexa Fluor 555 (Invitrogen Molecular Probes, Carlsbad, California, USA) for Stx2, Phage or GFAP immunofluorescence, and goat IgG anti-mouse/Texas Red (Amersham, GE, Piscataway, NJ, USA) for NeuN immunofluorescence. All secondary antibodies were prepared in the same buffer with 0.3% Triton X-100 and incubated for 2 h at room temperature. Finally, sections were rinsed with 10 mM PBS and mounted on slides. Controls were performed using the same procedure but without adding the primary antibody. A positive control was aggregated for the Stx2 inmunofluorescence; these brain sections were analyzed from Stx-treated mice (1 μg per mice injected intravenously with a final volume of 100 μl from a 0.01 μg/μl solution).

### Analysis of Micrographs

The mean data were obtained from the measurement of the micrographs of eight sections from four independent brains per treatment. A confocal laser scanning biological microscope (Olympus FV10-ASW) was used. The images obtained were analyzed using Image-J software (NIH, city, USA).

The “cell counter” plugin from Image-J software was employed to quantify the number of Stx2 immunoreactive cells and the abnormal NeuN. The criterion to determine the neuronal-stressed phenotype was the displacement of the nuclear marker to the cytoplasm. The ROI manager analyzer tool in Image-J software was employed to quantify the expression of GFAP and the presence of the bacteriophage, and to determine reactive astrocytes and whether the phages were present in the cerebral parenchyma. For this purpose, the color channels of the micrographs were split by the software, and the red color channel (now on an 8 bit gray scale format) was selected to analyze the area of interest. To observe in which CNS cells the phages were localized, the phage immunofluorescence assay was followed by that of GFAP (Astrocytes), CC1 (Oligodendrocytes), NeuN (neurons) and IBA-1 (microglia). Hoechst 33342 (Sigma, St. Louis, MO, USA) was used (1 μg/ml 10min) to show the cell nuclei in all immunofluorescence assays. The values from the background observed in all control micrographs of the anti-GFAP and anti-bacteriophage 933W were subtracted from the treated mouse micrographs.

### Statistical Analysis

The data are presented as mean ± SEM for Stx2 immunofluorescence. Statistical significance was performed using a one-way analysis of variance (ANOVA) followed by Tukey's multiple comparison test between the three tests (lysogenic C600Φ933W, C600 and positive control). In the case of the other three immunofluorescence assays (anti-bacteriophage 933W, NeuN and GFAP), where there were only two different treatments (*E. coli* C600ϕ933W and *E. coli* C600), statistical significance was performed using a *t*-test (Wilcoxon matched pair test) with a confidence interval of 95% (GraphPad Prism 4, GraphPad Software, Inc.). The criterion for significance was *p* < 0.05 for all the experiments.

## Results

### Growth Curves of *E. coli* C600 and *E. Coli* C600Φ933w

**Figure 1:**
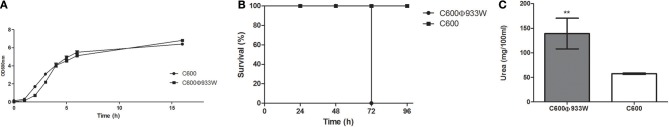
**(A)** Growth curves of *E. coli* C600 and *E. coli* C600Φ933W. Both strains present a similar growth curve with a non-significant difference between the viability counts. **(B)** Survival curve. Survival rates of mice infected with *E. coli* C600Φ933W are shown. Mice infected with *E. coli* C600 were used as controls. **(C)** Stx-induced renal damage. Plasmatic urea levels at 72 h post-infection were measured as a parameter of renal damage. Each bar represents the mean ± SEM of 4–6 mice/group. ^**^P = 0.0095.

In order to evaluate the growth parameters of the strains to be used in the infection assays, their growth curves were analyzed. Figure 1A shows that *E. coli* C600 (strain used as the negative control) and *E. coli* C600Φ933W (strain lysogenic for bacteriophage 933W) did not differ significantly between 0 and 15 h. The strains also showed a similar viability count at 16 h, when cultures were used to prepare the inoculums to infect mice.

### Mice Infected With *E. coli* C600Φ933w Died 72 h After Infection

Mice infected intragastrically with 2 × 10^7^ CFU/mouse non-pathogenic C600 strain lysogenized with bacteriophage 933W (C600Φ933W) died 72 h post-infection (Figure 1B). Control mice infected with non-pathogenic *E. coli* strain C600 remained healthy for 5 days, after which they were sacrificed.

Mice infected with *E. coli* C600Φ933W showed a significant increase in the plasma urea level compared to mice infected with *E. coli* C600 (Figure 1C), demonstrating kidney damage due to bacteriophage induction and Stx production.

### Histology of Kidneys and Intestines of Mice Infected With *E. coli* C600Φ933w

In another set of experiments, groups of mice were infected with *E. coli* C600Φ933W or C600 and sacrificed at 72 h. Kidneys, intestines and brains of the mice were excised for histological studies.

**Figure 2:**
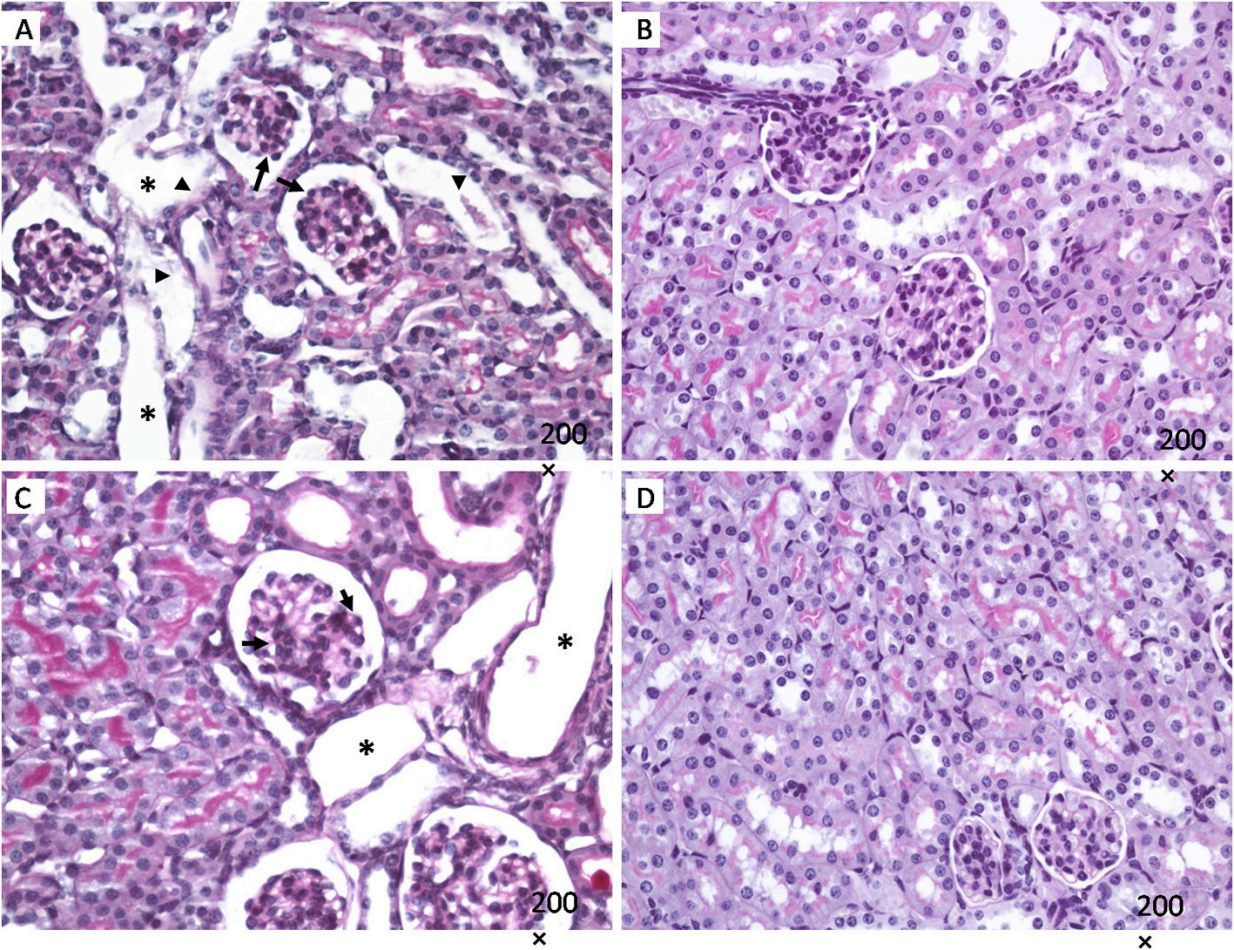
Kidney histology. Mesangial proliferation is observed in in mice infected with *E. coli* C600Φ933W (black arrow in **A,C**), as well as necrosis and detachment of tubular cells (black arrowhead in **A,C**), and tubular dilation (black asterisk in **A,C**). No effect was observed in mice infected eith the control strain *E. coli* C600 **(B,D)**. Periodic acid shift (PAS). Magnification: 200 ×.

Kidneys from mice infected with *E. coli* C600Φ933W showed mesangial proliferation (black arrow in Figures 2A,C), necrosis and detachment of tubular cells (black arrowhead in Figures 2A,C), as well as tubular dilation (black asterisk in Figures 2A,C). Control mice infected with *E. coli* C600 did not show kidney damage (or presented normal features) (Figures 2B,D).

**Figure 3:**
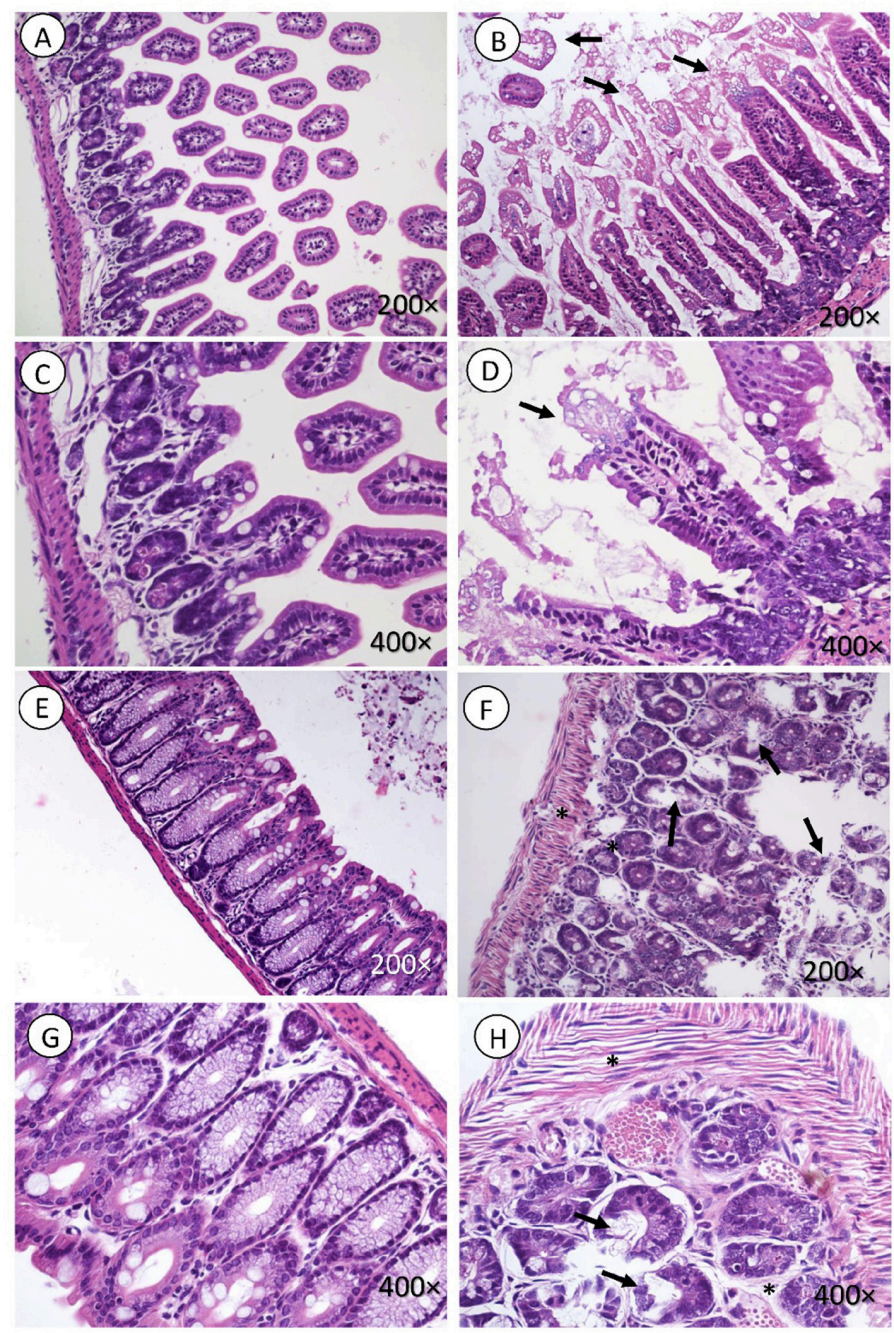
Intestine histology. Necrosis is observed in intestinal microvilli (black arrow, **B,D**) of the small intestine and in the crypt of the large intestine (black arrow in **F,H**). There is also marked edema surrounding the crypts of the mucosa and muscle fibers (black asterisk in **F,H**). **(A,C,E,G)** corresponding to the control group, did not present lesions. H&E. Magnification: (**A,B** and **E,F** 200 ×, **C,D** and **G,H**) 400 ×.

Intestines of mice infected with *E. coli* C600Φ933W showed signs of damage, including lymphocytic infiltration in the muscle, submucosal and mucosal edema in the small intestine (Figure 3), and mucosal edema in the large intestine (Figures 3F,H). Control mice infected with *E. coli* C600 did not show intestinal damage (Figures 3A,C,E,G).

### Immunofluorescence Analysis of Stx2 Expression in Brains

**Figure 4:**
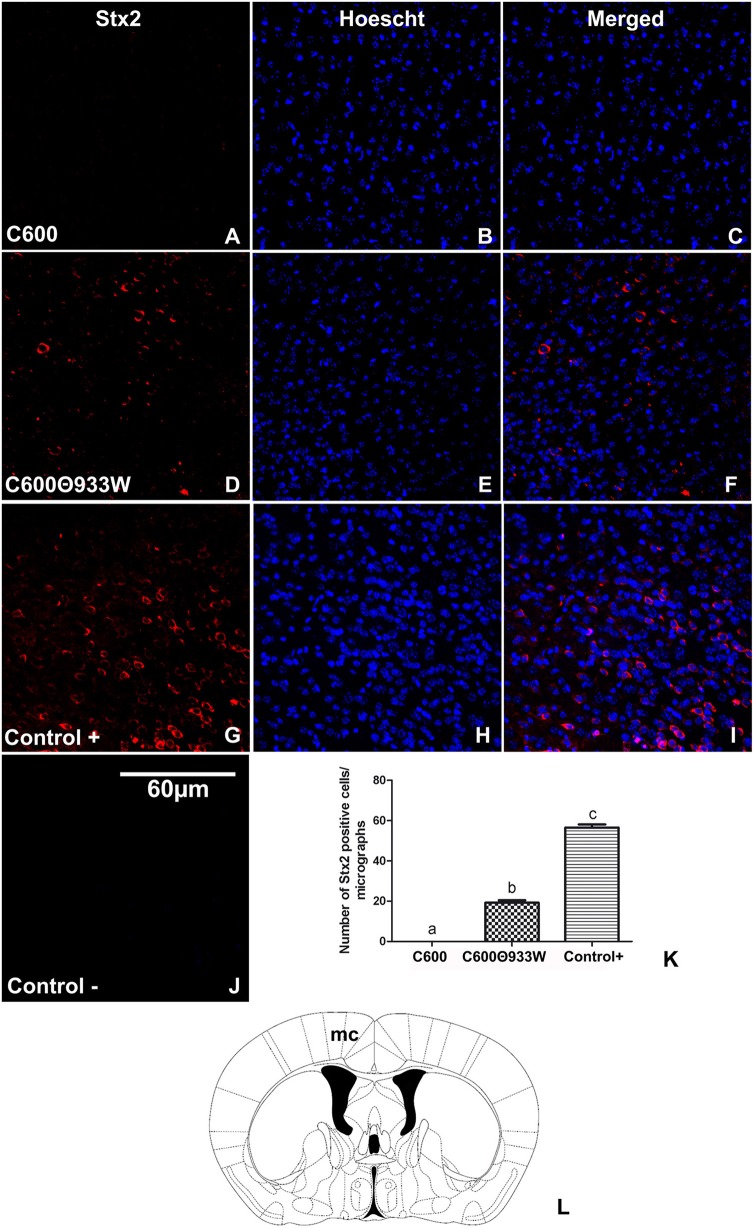
Detection of Stx2 in brains of mice infected with *E. coli* C600Φ933W. **(A–C)** A representative brain section from control mouse infected with *E. coli* C600. **(D–F)** Brain section from mouse infected with *E. coli* C600Φ933W. **(G–I)** Brain section from a positive control mouse after 4 days of treatment with 1 μg of Stx2. **(J)** Negative control. **(K)** Number of Stx2-immunopositive cells in in the mouse motor cortex (mc) per micrograph. Stx2 immunopositive cells are indicated by arrows in **(F,I)**. **(L)** The area observed is located in the mouse mc. The scale bar in “**J**” applies to all micrographs.

To evaluate the presence of Stx2 in brains from *E. coli* C600Φ933W-infected mice, an immunofluorescence assay using a polyclonal anti-Stx2 antibody was performed (Tironi-Farinati et al., 2010). The analyzed area was the motor cortex (Figure 4L). Immunopositive cells for Stx2 were not found in control mice infected with *E. coli* C600 (Figures 4A–C), but were observed in *E. coli* C600Φ933W-treated mice (Figures 4D–F). Also, positive control mice treated with 1μg of Stx2 showed a higher number of Stx2-immunopositive cells (Figures 4G–I) compared with *E. coli* C600Φ933W-treated mice (17.73 ± 1.89 C600Φ933W vs. 56.6 ± 2.46 positive control cells, Figure 4K). Stx2 was not immunodetected in the negative controls (Figure 4J).

### Immunofluorescence Analysis of Bacteriophage Detection in Brains

After Stx2 was immunodetected in the brain, the presence of the bacteriophage containing the sequence for Stx2 was further determined by immunofluorescence, employing an in-house polyclonal antibody against bacteriophage 933W. Additionally, the antibodies anti-CC1, anti-GFAP, anti-NeuN and anti-IBA-1 were used to identify oligodendrocytes, astrocytes, neurons and microglia, respectively, to determine which cell type was immunolabeled with the anti-phage 933W.

**Figure 5:**
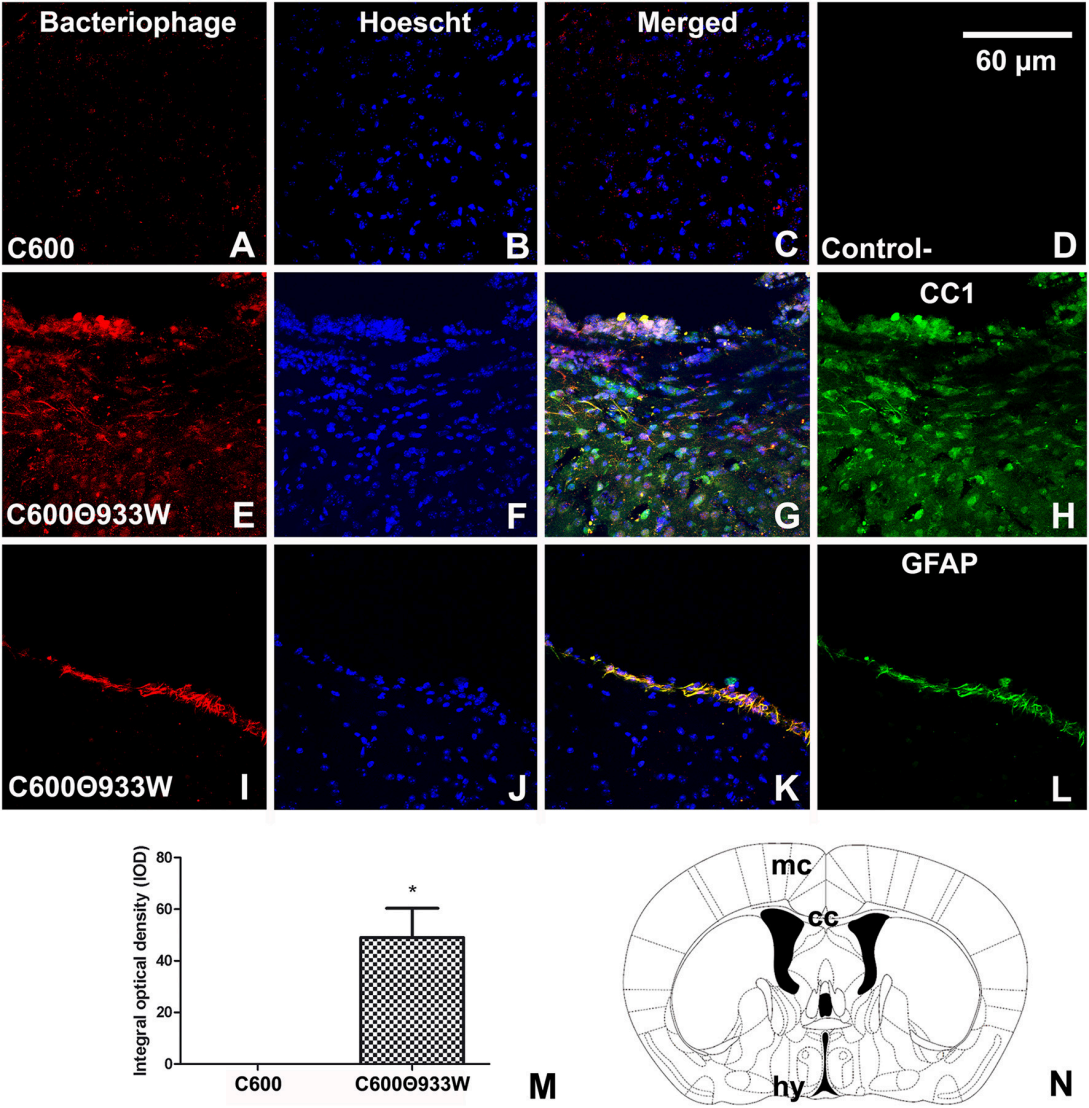
Bacteriophage detection in brains of mice infected with *E. coli* C600Φ933W. **(A,C,D)** Brain from control mouse infected with *E. coli* C600. **(E,L)** Brain from mouse infected with *E. coli* C600Φ933W 72 h after inoculation. **(E–H)** External capsule; **(I–L)** hypothalamus. **(B)** Negative control omitting the secondary antibody. **(N)** Bacteriophage were detected in the mouse corpus callosum (cc) and hypothalamus (hy) (the red square shows where the micrographs were taken). **(M)** Quantification of bacteriophages in the cerebral parenchyma by integral optical density (IOD). lv, lateral ventricle; gl, glia limitans. The scale bar in “**B**” applies to all micrographs.

Accordingly, bacteriophage 933W was immunolocalized in the cerebral parenchyma (Figure 5N) in oligodendrocytes in the white matter of the corpus callosum (Figures 5E–H), and in GFAP-immunoreactive astrocytes forming the glia limitans of the ventral hypothalamus (Figures 5I–L). Finally, bacteriophage 933W was not observed in mice infected with *E. coli* C600 (Figures 5A,C,D) or in negative controls omitting the primary antibody (Figure 5B). The presence of bacteriophage 933W in the cerebral parenchyma was quantified: 0.00 C600 vs. 40.051 ± 1.89 C600Φ933W AU in IOD (Figure 5M).

### Bacteriophage 933W Increased the Expression of GFAP in Astrocytes

**Figure 6:**
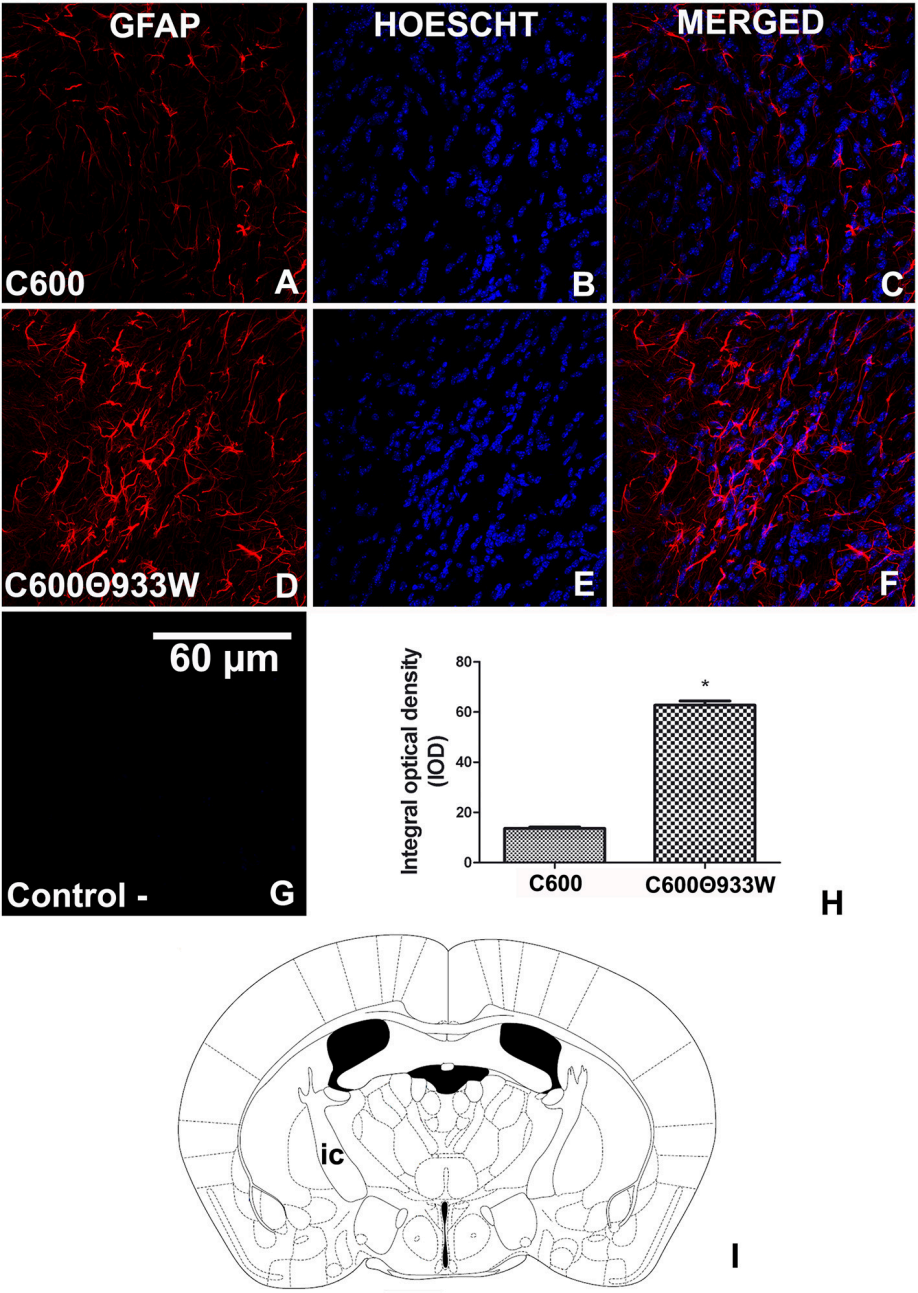
GFAP expression increased in astrocytes after *E. coli* C600Φ933W infection. **(A–C)** Brain section from control mouse infected with *E. coli* C600. **(D–F)** Brain section from mouse infected with *E. coli* C600Φ933W. **(G)** Negative control omitting the secondary antibody. **(H)** GFAP expression measured by integral optical density (IOD). **(I)** the area observed is located in the mouse internal capsule (ic). The scale bar in “**G**” applies to all micrographs. ^*^P < 0.005 as calculated by Student's *t*-test.

We have previously demonstrated that mammalian cells are able to express Stx2 after prokaryotic plasmid (pStx2) *in vivo* transfection, which led to an increase of GFAP in astrocytes (Bentancor et al., 2013). Now, bacteriophage 933W was induced from *E. coli* C600Φ933W *in vivo* to determine whether it could produce these reactive astrocytes, which are associated with brain damage. GFAP was significantly up-regulated in the internal capsule (Figure 6I) of mice infected with *E. coli* C600Φ933 72 h post-infection (Figures 6D–F) compared with the *E. coli* C600 treated-mice (Figures 6A–C) (13.63 ± 2.209 C600 vs. 62.77 ± 9.102 C600Φ933W AU in IOD, Figure 6H). No immunofluorescence corresponding to GFAP was found in negative controls omitting the primary antibody (Figure 6G).

### Bacteriophage *E. coli* C600Φ933w Produced Neuronal Damage

**Figure 7:**
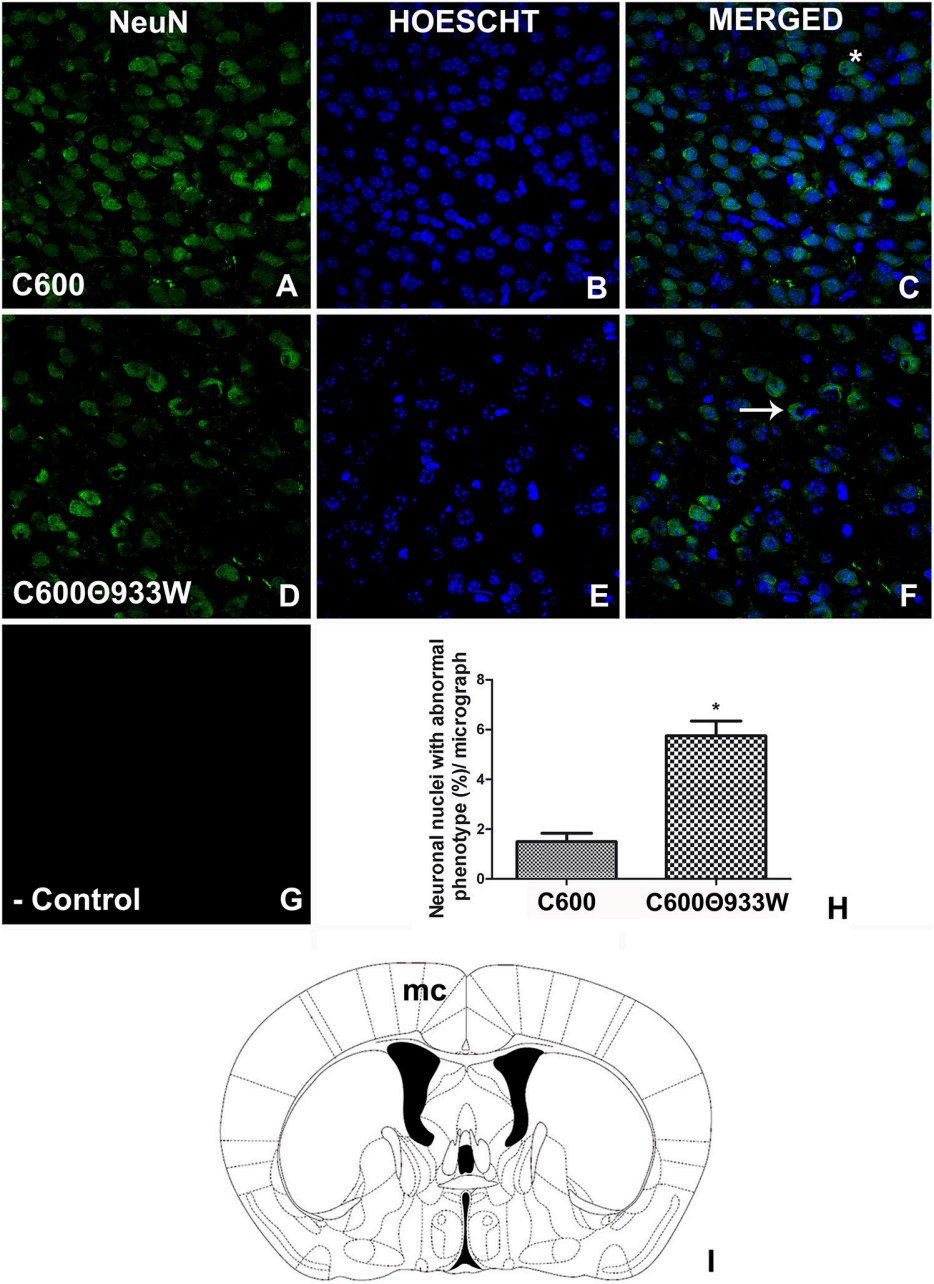
*E. coli* C600Φ933W infection produced a NeuN damage pattern in the mouse brain neurons. **(A–C)** Brain section from control mouse infected with *E. coli C600*. **(D–F)** Brain section from mouse infected with *E. coli* C600Φ933W. **(G)** Negative control omitting the secondary antibody. **(H)** NeuN quantification showing a significant increase in the amount of abnormal cellular NeuN immunolocalization in *E. coli* C600Φ933W-treated mice (arrow in **F**) compared with a conserved and homogeneous nuclear immunofluorescence pattern in *E. coli* C600-treated mice (asterisk in **C**). **(I)** the area observed is located in the mouse motor cortex (mc). The scale bar in “**G”** applies for all micrographs. ^*^P < 0.005 as calculated by Student's *t*-test.

It is known that Stx2 induces toxicity in neurons (Fujii et al., 1994; Goldstein et al., 2007). The neuronal nuclear marker NeuN was used to determine whether bacteriophage 933W caused toxicity in neurons. The criterion used to identify damaged neurons was the ectopic cellular sub-localization of NeuN (Pinto et al., 2013). A conserved and homogeneous nuclear immunofluorescence pattern for NeuN confirmed healthy neurons in control mice infected with *E. coli* C600 (Figures 7A–C). In contrast, a significant neurodegenerative perinuclear or cytoplasmic immunofluorescence pattern was observed in *E. coli* C600Φ933W-treated mice (Figures 7D–F) in comparison with the conserved neurons of *E. coli* C600 (1.53 ± 0.42 C600 vs. 5.68 ± 0.66 C600Φ933W cells). No immunofluorescence corresponding to NeuN was observed in negative controls omitting the primary antibody (Figure 7G).

## Discussion

Shiga toxin-producing *E. coli* has long been recognized as a principal cause of HUS (Richardson et al., 1988; Karmali, 1989). More recently, the induction of Stx-encoding bacteriophages inserted into the STEC genome was found to be critical for HUS development in an EHEC mouse model (Tyler et al., 2013). Other studies have reported that bacteriophage induction can be enhanced by commonly consumed drinks (Pierzynowska et al., 2018) and inhibited by phenethyl isothiocyanate (Nowicki et al., 2014).

The aim of the present work was to analyze if Stx-encoding bacteriophages can cause renal and intestinal damage in absence of other STEC pathogenicity factors. We therefore used the C600Φ933W lysogen, because *E. coli* C600 does not have proteins implicated in intestinal colonization and damage. The mouse model described here is based on one previously developed by Dr. Palermo's laboratory to analyze renal and intestinal damage and lethality, using the wild type O157:H7 (EDL933) *E. coli* strain (Fernandez-Brando et al., 2014). Mice infected with non-pathogenic *E. coli* carrying only a Stx-producing bacteriophage (*E. coli* C600Φ933W) developed renal, intestinal and brain damage typical of Stx toxicity after STEC infection. It was thus demonstrated that a pathogenic bacterial background is not necessary for the development of physiological symptoms typical of HUS.

In this study, antibiotics were used to cause bacteriophage 933W induction and replication, and Stx production and release (Zhang et al., 2000; Wagner et al., 2002). Bacteriophage induction *in vivo* using antibiotics has been previously reported by our team (Amorim et al., 2014). Here, we did not include a control without an inducing agent, because high levels of spontaneous induction (understood as induction in the absence of an inducing agent) (Livny and Friedman, 2004) of bacteriophage Φ933W have been described *in vitro* in an *E. coli* C600Φ933W lysogen (Imamovic and Muniesa, 2012). Therefore, the control used was the *E. coli* C600 strain without bacteriophage 933W.

Strikingly, a lethal response of mice infected with *E. coli* C600Φ933W was observed after bacteriophage induction. To determine whether death was related to Stx, we analyzed typical physiological alterations triggered by STEC infection, including an increase in urea level, and intestinal, kidney and brain damage. STEC infections produce kidney damage, with an increase in urea level and glomerular and tubular abnormalities (Zotta et al., 2008). Both these alterations corresponding to Stx toxicity were observed in mice infected with *E. coli* C600Φ933W. In addition, intestinal and brain damage was studied by histological and immunohistochemistry assays. Edema was observed in small and large intestines, as previously reported in mice after STEC infection (Zotta et al., 2008).

The absence of bacterial pathogenic factors in the *E. coli* C600 carrying Φ933W used in this work allows us to hypothesize that the damage observed could be associated with free bacteriophages infecting gut bacteria, which drove bacteriophage replication and Stx production.

We previously reported that mice injected with a Stx-expressing plasmid developed CNS injury, showing upregulation of GFAP expression and a decrease in NeuN expression (Bentancor et al., 2013). The damage observed in the current work was similar, but provoked by Stx expressed by the bacteriophage in absence of a pathogenic bacterial background. Stx and bacteriophages were detected in brains by immunohistochemistry. A recent study found bacteriophage T4 was internalized by human brain microvascular endothelial cells (hBMec) (Nguyen et al., 2017). Here we report for the first time the presence of bacteriophages in the brain *in vivo* after intragastrical infection with a non-pathogenic strain.

In conclusion, the results of this work show that Shiga toxin-expressing bacteriophages (STB), in absence of other pathogenic bacterial factors, provoked intestinal, renal and brain damage associated with HUS development. We have previously reported that chitosan (Amorim et al., 2014) and cationic peptides (Del Cogliano et al., 2017) have the capacity to inhibit STB as a therapeutic alternative for HUS infection. The new animal model described here is proposed as a useful tool to study bacteriophages as a therapeutic target, independently of the bacterial background, and opens an innovative approach to treating infections caused by toxin-encoding bacteriophages.

## Author Contributions

LB designed, analyzed the data and wrote the manuscript. MP provided advice on experimental design, data interpretation, obtained funding and critical reading of the manuscript. MD, AP, FO, RF-B, MM, and PG performed experiments and provided advice on experimental design, JG, EZ, and PG provided advice on experimental design.

### Conflict of Interest Statement

The authors declare that the research was conducted in the absence of any commercial or financial relationships that could be construed as a potential conflict of interest.
